# Principal Aspects Regarding the Maintenance of Mammalian Mitochondrial Genome Integrity

**DOI:** 10.3390/ijms18081821

**Published:** 2017-08-22

**Authors:** Panagiotis V. S. Vasileiou, Iordanis Mourouzis, Constantinos Pantos

**Affiliations:** 1 Department of Basic Medical Sciences, Laboratory of Histology & Embryology, School of Medicine, National and Kapodistrian University of Athens, 75 MikrasAsias Avenue, Goudi, Athens 11527, Greece; panagiotis.vasileiou@yahoo.gr; 2 Department of Pharmacology, School of Medicine, National and Kapodistrian University of Athens, 75 MikrasAsias Avenue, Goudi, Athens 11527, Greece; imour@med.uoa.gr

**Keywords:** genomic instability, mitochondrial genome, nucleoids, mitochondrial DNA repair mechanisms, heteroplasmy, fusion, fission, mitophagy

## Abstract

Mitochondria have emerged as key players regarding cellular homeostasis not only due to their contribution regarding energy production through oxidative phosphorylation, but also due to their involvement in signaling, ion regulation, and programmed cell death. Indeed, current knowledge supports the notion that mitochondrial dysfunction is a hallmark in the pathogenesis of various diseases. Mitochondrial biogenesis and function require the coordinated action of two genomes: nuclear and mitochondrial. Unfortunately, both intrinsic and environmental genotoxic insults constantly threaten the integrity of nuclear as well as mitochondrial DNA. Despite the extensive research that has been made regarding nuclear genome instability, the importance of mitochondrial genome integrity has only recently begun to be elucidated. The specific architecture and repair mechanisms of mitochondrial DNA, as well as the dynamic behavior that mitochondria exert regarding fusion, fission, and autophagy participate in mitochondrial genome stability, and therefore, cell homeostasis.

## 1. Introduction

Owing to the symbiotic relationship that mitochondria and eukaryotic cells established about 1.5 billion years ago, mammalian mitochondrial biogenesis and function require the coordinated action of two genomes: nuclear and mitochondrial [1]. Both nuclear genes (between 1000 and 2000 genes) and thousands of copies of the maternally inherited mitochondrial DNA (mtDNA) genes participate in the assembly of these multifaceted organelles [2]. Nuclear genes code for the vast majority of mitochondrial proteins which are located within the organelle, as well as regulating factors regarding cellular bioenergetics. Human mtDNA genes code for 13 critical polypeptides that are involved in oxidative phosphorylation, plus the 22 transfer ribonucleic acids (tRNAs) and two ribosomal RNAs required for their expression. None of the polypeptides required for replication, transcription, and repair of mtDNA, or for processing and translation of the mitochondrial RNA (mtRNA) transcripts are encoded by the mtDNA [3]. Surprisingly, this bi-genomic organization has been retained through evolution, and it seems to serve bioenergetic benefits [4].

MtDNA is the cornerstone regarding energy production through oxidative phosphorylation since it encodes components of four out of five mitochondrial respiratory complexes [5]. Indeed, advances in molecular and cell biology, as well as in genetics and biochemistry gave us the opportunity to understand that mitochondria are much more than the powerhouses of the cells, exerting a role in crucial cellular functions, including gene expression, ion homeostasis, and cell death signaling [6,7]. Therefore, the integrity of mtDNA is crucial not only for optimal substrate utilization and energy production, but also for cell homeostasis in general.

Due to their involvement in reactive oxygen species (ROS) production, mitochondria are both a major source and, at the same time, targets of genotoxic stress [8,9]. Instability regarding nuclear genome has been extensively studied; however, the importance of mitochondrial genome integrity has only recently begun to be elucidated [10,11]. Herein, we are summarizing basic principles of mechanisms regarding the integrity of the mitochondrial genome. Our approach has been stratified in three levels: (1) defensive mechanisms against mtDNA damage before the genotoxic insult, mostly referring to the specific organization of mitochondrial genome, and in particular mitochondrial nucleoid machinery and associated proteins, as well as other principal regulators of mitochondrial biogenesis;(2) when the DNA damage occurs, referring to DNA repair mechanisms that function efficiently within mitochondria; and (3) when mtDNA repair systems fail to deal with the DNA damage, referring to heteroplasmy and mitochondrial dynamic processes of fusion, fission, and mitochondrial autophagy.

## 2. Organization of Mitochondrial Genome

The mitochondrial genome was discovered about 60 years ago, after the pioneering studies of Nass and Schatz [12,13]. Human mtDNA comprises 16,569 bp of closed-circular double-stranded DNA [14]. The circularity of mtDNA does not refer to all eukaryotic organisms; accumulating evidence suggest that most of mtDNA in fungi and plants is linear in form, suggesting that the circular DNA found in animal mitochondria is an exception rather than the rule [15].

MtDNA contains a regulatory region, the displacement-loop (d-loop) region, which contains initiation sites for transcription and replication by the mitochondria-specific polymerase-γ (pol γ); pol γ is the only replicative DNA polymerase known to function within mitochondria [16,17]. Both replication and repair of mtDNA are dependent on this nuclear-encoded DNA polymerase [18]. Pol γ holoenzyme is actually a heterotrimer comprised of two accessory subunits (p55) and one pol γ catalytic subunit (p140); the accessory subunits are encoded by the *POLG2* gene, whereas *POLG* encodes the catalytic subunit [18]. It is noteworthy that the isolated pol γ catalytic subunit exerts a remarkable fidelity of DNA replication, owing to its high nucleotide selectivity and exonucleolytic proofreading, but the inclusion of the p55 subunits reduces fidelity of replication by promoting extension of mismatched DNA termini [19]. Other major proteins involved in replication of mtDNA, together with the pol γ enzyme, are the mitochondrial DNA helicase, Twinkle, and the mitochondrial single stranded DNA binding protein (mtSSB) [20].

Over 200 mutations in *POLG* have been associated with numerous mitochondrial diseases [21,22,23]. The consequences of these mutations include alterations in the assembly of the replication apparatus, reductions of the enzymatic activities of the polymerase and helicase or the fidelity of DNA replication, as well as disruption of the polymerase holoenzyme [18]. Nevertheless, the frequency of these mutations is unusual. Surprisingly, it has been demonstrated, based on various mouse models with disrupted pol γ exonuclease activity, that mutations eliminating exonuclease function of pol γ are not embryonically lethal [24,25,26]. However, pol γ variants with decreased exonuclease activity targeted specifically to the heart proved to cause severe cardiomyopathy along with accelerated mtDNA mutagenesis [23]. Similarly, mice expressing defective mtDNA polymerase exhibited characteristics of premature aging, along with enlarged hearts [25,26].

Twinkle helicase is essential for mtDNA maintenance and a key regulator of mtDNA copy number; inhibition of Twinkle expression in cultured cells resulted in rapid mtDNA depletion, whereas overexpression of Twinkle in transgenic mice led to considerable increase in mtDNA copy number [27]. Dominant mutations of Twinkle are associated with multiple mtDNA deletions in post-mitotic tissues (namely, in the brain, the skeletal muscle, and the heart), whereas recessive mutations of Twinkle cause mtDNA depletion in the brain and liver; unfortunately, the molecular mechanistic basis of the tissue specificity regarding mtDNA maintenance disorders has not yet been elucidated [20,28,29,30,31,32,33]. Interestingly, Twinkle disorders mimic those of primary deoxynucleoside triphosphates (dNTP) pool defects [34]. According to Nikkanen et al, the mitochondrial replication machinery communicates with cytoplasmic dNTP pools and upregulation of glutathione synthesis through glucose-driven de novo serine biosynthesis contributes to the metabolic stress response; mtDNA depletion disorders show low and imbalanced dNTP pools, whereas multiple mtDNA deletion disorders show imbalanced and high dNTP pools [34].

Transcriptional control of mitochondrial biogenesis involves a number of regulators such as the mitochondrial transcription factor A (TFAM), nuclear respiratory factors 1 and 2 (NRF 1/2), and peroxisome proliferator-activated receptor gamma co-activator (PGC1a) [35,36,37]. TFAM is a nuclear-encoded transcription factor that binds to a common upstream enhancer of the promoter sites of the two mitochondrial DNA strands [37]. Of note, cardiac-specific TFAM knockout mice displayed a progressive cardiomyopathy phenotype of cardiac hypertrophy, with decreased levels of mtDNA and an accompanying severe decline of respiratory chain enzyme activities along with a decreased mitochondrial ATP production rate [38]. NRF 1/2 control many, but not all of the mitochondrial genes [39]. They are both recognized by TFAM, thus mediating the coordinated activation of mitochondrial and nuclear genome during mitochondrial biogenesis [37]. The primary regulator of the coordinated transcription of mitochondrial and nuclear genomes is the PGC1a [37,40]. PGC-1a is enriched in tissue with high oxidative activity-like the heart- and it is rapidly induced under conditions of enhanced energy demand [41,42].

In contrast to the nuclear genome which is compacted and organized by histone octamers, mtDNA is devoid of nucleosomes and organized as protein-DNA complexes known as nucleoids (mitochondrial nucleoids/mt-nucleoids) [3,43]. The precise structure of these nucleoids in which mtDNA is organized is not fully elucidated. Initially, it was believed that each nucleoid carries six–10 copies of mtDNA, but later on, it has been established with super-resolution imaging techniques that each nucleoid may contain only one or two (on average, 1.4) molecules of mtDNA, or according to other findings, three mtDNA genomes [44,45]. This special molecular organizing unit of mitochondrial DNA serves as a mechanism of protection, maintenance and accurate propagation of the mitochondrial genome; disruption in any component of this assembly results inmtDNA deletionsand mutations [43].Measurements of the absolute mtDNA copy number per cell and per nucleoid using stimulated emission depletion (STED) and photo activating light microscopy (PALM) imaging studies revealed that mtDNA, compared with bacterial nucleoid or nuclear chromatin, exerts a greater DNA packaging density, in regard with nucleoid volume, nuclear volume, and genome size [44,46]. The clinical significance of the tight package of mtDNA is associated with the protection against mutagenesis [3].

Several proteins are involved in packaging mtDNA in the form of mt-nucleoids. The best-characterized and most abundant protein binding duplex mtDNA, is theTFAM [47]. TFAM has a dual role in the maintenance of mtDNA, through both transcription and nucleoid formation [48]. It enhances mtDNA transcription in a promoter-specific fashion in the presence of mtRNA polymerase and transcription factor B [49,50]. In addition, TFAM exerts DNA-binding properties; approximately 900 molecules of TFAM are bound to one molecule of mtDNA [51].Depletion of *TFAM* gene resulted in embryonic lethality while tissue-specific disruption of *TFAM* gene in heart and muscle led to a mosaic cardiac-specific progressive respiratory chain deficiency, dilated cardiomyopathy, and atrioventricular heart conduction blocks and proved lethal after two to four weeks of age [52,53].

Other components of nucleoids include the Twinkle helicase, the mitochondrial polymerase γ, as well as mtSSB protein, all of which have been identified as participants in mtDNA maintenance [54]. The array of proteins found associated with nucleoids further includes helicases and RNA binding proteins, chaperones, and quality control proteases, mitochondrial ribosomal proteins as well as lipid metabolic enzymes [3]. According to Bogenhagen, many of these elements may represent contaminants in nucleoid preparations during proteomic analysis, however one cannot exclude the possible functional role of these proteins regarding mitochondrial biogenesis [3]. Among these potential nucleoid-associated proteins are included prohibitin, ATPase family AAA domain-containing protein 3(ATAD3), and p53.Except for the former, the other two (ATAD3 and p53) have not been detected in nucleoid purifications [55].

Prohibitin (PHB) proteins (PHB1 and PHB2) belong to chaperones’ system and reside in the intermembrane space [56]. Prohibitins’ primary function is to stabilize newly synthesized polypeptides in mitochondria, as they serve as foldase-unfoldase molecular chaperones; importantly, prohibitins exert various intra-mitochondrial as well as extra-mitochondrial effects, both of which are implicated in mitochondrial DNA stability [57,58]. PHB1 maintains the organization of the mt-nucleoids through a TFAM-independent pathway or through an undefined nucleoid factor (Factor X); alternatively, PHB1 maintains the copy number of mtDNA through a TFAM-dependent pathway [59]. In addition, PHB1 contributes to the organization of the inner membrane via an interaction with Opa1 (optic atrophy protein 1), a dynamin-related GTPase involved in mitochondrial fusion, cristae organization, and control of apoptosis [60]. Moreover, PHB2 regulates the stability of mitochondrial proteins such as HS1-associated protein X-1 (Hax-1) and Opa1, and is involved in anti-apoptosis and regulation of mitochondrial morphology [61]. Of note, mutations (mis-sense point mutations) in *Opa1 gene* lead to genomic instability through mtDNA deletions [62,63,64]. Furthermore, prohibitins exert various extra-mitochondrial cellular functions including roles in apoptosis, cell cycle regulation, transmembrane signal transduction, and life span regulation [58,65].

ATAD3 belongs to the family of the AAA-ATPases (ATPases associated with diverse cellular activities). Human ATAD3 refers to two mitochondrial paralogs, ATAD3A and ATAD3B [66]. It is considered to be an inner membrane mitochondrial protein; however, C-terminus islocated in the mitochondrial matrix and could be associated with the nucleoid, possibly through an indirect manner [55,67]. In vitro, ATAD3 has been shown to bind directly to the D-loop of mtDNA [68]. The implication of ATAD3 in the mt-nucleoid organization may also be related to the exact localization of this protein at contact points where the outer and the inner membranes of mitochondria are juxtaposed; these contact points are in relation to contact areas between the endoplasmic reticulum and mitochondria and serve as transit zones for many molecules such as neo-synthesized proteins, calcium and lipids [67,69,70]. It has been demonstrated that ATAD3 deficiency destabilizes mt-nucleoid organization through the dissociation of mtDNA fragments which are held together by this protein [71]. Furthermore, ATAD3 plays a critical role in cellular cholesterol homeostasis; ATAD3 deficiency causes aberrant mtDNA organization and is associated with elevated free cholesterol and increased expression of genes involved in cholesterol metabolism [72]. Importantly, it seems that perturbed cholesterol metabolism affects both replication and segregation of mtDNA [72]. In addition, de novo mutations in ATAD3A were recently found to cause a neurological syndrome with developmental delay, hypotonia, spasticity, optic atrophy, axonal neuropathy, and hypertrophic cardiomyopathy [66].

P53 is a soluble predominantly nuclear protein; in the nucleus, p53 functions as a transcription factor through a specific DNA-binding domain, but also represses gene expression through an interaction with histone deacetylases [73]. A small fraction of the total cellular amount of nuclear transcription factor p53 seems to be located at and within mitochondria. Recent evidence suggest that after genetic stress or death signals, a fraction of p53 translocates into the mitochondria, where it acts in a “biphasic” manner, either inducing or attenuating apoptosis progression.P53 is localized to the inside face of the inner membrane i.e., in the matrix, in which mtDNA is also located [74,75]. Within the human mitochondrial genome a p53-recognition motif has been recognized [76]. Mitochondrial p53 also exerts physical interactions with mitochondria DNA and DNA polymerase γ and enhances error correction activities [77]. Furthermore, it has been demonstrated that death signals can induce its translocation to the mitochondria, whereas p53 was shown to physically and functionally interact with both, the mtDNA and pol γ in response to mtDNA damage induced by exogenous and endogenous insults [78].

Given that contrary to what is happening regarding the nuclear genome, the mtDNA replication process is not restricted to the S phase of cell cycle, two models have been proposed to explain how mitochondrial nucleoids mediate mtDNA inheritance. The “faithful nucleoid model”, proposed by Jacobs, supports that individual nucleoids replicate only their own exact genetic content, thus mtDNA content within a nucleoid is generally static and nucleoids do not exchange mtDNAs between each other [79]. On the opposite site, D’ Aurelio proposed the “dynamic nucleoid model” according to which nucleoids are subject to remodeling and have a dynamic organization allowing nucleoids to exchange mtDNAs [80]. Current evidence supports that mitochondrial nucleoids tightly regulate their genetic content rather than freely exchanging mtDNAs, in agreement with the faithful nucleoid model [81].

## 3. Mitochondrial DNA Repair Machinery

Both environmental and intrinsic genotoxic insults constantly threaten the integrity of nuclear as well as mitochondrial DNA [82]. Exogenous factors include environmental insults such as ionizing radiation, ultraviolet (UV) radiation, and alkylating agents. Endogenous noxious agents include reactive oxygen species and products generated as a consequence (e.g., lipid peroxides), endogenous reactive chemicals (e.g., aldehydes and *S*-adenosylmethionine), and chemical DNA instability (e.g., deamination of bases, depurination or depyrimidination) [83]. Of note, the vast majority of mutations in human tissues are certainly of endogenous origin [74]. There are six main types of mtDNA damage: alkylation damage, hydrolytic damage, formation of adducts, mismatched bases, DNA strand breaks, and oxidative damage [84].

It is generally believed that mtDNA, compared with nuclear DNA, is more prone to damage; indeed, it has been estimated that mtDNA has 10–20 times higher mutation rate than the nuclear genome [85,86]. According to Yakes and Van Houten, several factors contribute to the increased susceptibility of mitochondrial genome to damage, such as the organization of mtDNA in nucleoids instead of chromatin, the limited DNA repair machinery of mtDNA, the presence of localized metal ions that may function as catalysts in the generation of ROS, or the propagation of mitochondrial damage through the generation of secondary ROS due to impairment of the electron transport chain and/or lipid peroxidation [87]. Importantly, recent studies, based on newer analytical techniques, support that specific oxidative lesions in specific tissues are less prevalent in mtDNA compared with nuclear DNA [88,89]. Experimental findings demonstrating that on some occasions, histones may enhance rather than reduce DNA damage, plus the possible protective role of mtDNA associated proteins, such as TFAM, which may serve as a shell for mtDNA, further challenge the belief about the vulnerability of mitochondrial genome [84].

Oxidative damage occurs basically from the generation of ROS within mitochondria; however, oxidative damage can also be caused by reactive nitrogen species, which are also present within the cell and their byproducts are able to oxidize or deaminate DNA bases and induce strand breaks [90,91]. Oxidative damage is the most common mechanism of damage as far as mitochondria are concerned. The first reaction against the potential genotoxic effect of ROS refers to antioxidant compounds and activities that are available within mitochondria, such as enzymatic antioxidants (i.e., catalase, glutathione peroxidase, thioredoxin, peroxiredoxin, and aldehyde dehydrogenase 2) and non-enzymatic antioxidants (i.e., glutathione, N-acetyl cysteine, ubiquinol, α-tocopherol, ascorbic acid, and lipoic acid) that have been shown to migrate into mitochondria due to oxidative stress [92,93]. During oxidative phosphorylation 1–2% of the oxygen that is consumed within the cell is released from mitochondria as ROS [94]. Under normal conditions, this amount of produced ROS results in low level of damage to mtDNA, which is rapidly repaired; however, during periods of increased and prolonged ROS exposure the generation of ROS leads to more extensive and persistent damage of mtDNA [87]. In any case, the proximity of mtDNA to the inner mitochondrial membrane where the electron transport chain constantly produces ROS as well as the mitochondrial membrane potential which facilitates the accumulation of lipophilic mutagenic cations, create a condition of susceptibility for mitochondrial DNA [95,96].

The maintenance of mitochondrial genome integrity is also supported by detection and repair mechanisms of DNA lesions [97]. Until recently, mitochondria were considered to exert a limited availability of DNA repair mechanisms, contrary to the several repair mechanisms that are definitely present in the nucleus; however, recent evidence suggest that some of the nuclear DNA repair pathways also function in mammalian mitochondria [9]. As mentioned above describing mt-nucleoid, a number of proteins have been reported to be localized in close proximity with the mtDNA assembly (such as RNA helicases, mitochondrial ribosomal proteins, chaperones, quality control proteases, and lipid metabolic enzymes) implicating their possible access to mtDNA following DNA damage [3,98]. Literally speaking, although mitochondria dispose of a competent and coherent base excision repair (BER) system, they lack effective mismatch repair (MMR) and completely lack nucleotide excision repair (NER) systems [20].

BER is the predominant DNA repair pathway in mitochondria; it is responsible for the removal of DNA bases altered by oxidation, alkylation, or deamination, and the repair of abasic sites and single-strand breaks resulting from spontaneous hydrolysis and oxidation, respectively [9,99]. ROS-induced oxidative lesions are preferentially eliminated by BER [84]. Of note, the repair of 8-hydroxyguanine (8-OHG or 8-oxoG), a major oxidative DNA base lesion which also serves as a biomarker of oxidative stress, is more efficient in the mitochondrial DNA than in the nuclear DNA [100,101]. It was initially believed that only short-patch BER (insertion of a single nucleotide) occurs in mitochondria, but recent evidence suggests that long-patch BER (insertion of a short sequence of two to six nucleotides) activity is present too [102,103,104,105,106]. Interestingly, the assembly of nuclear BER machinery needs a specific protein (XRCC1) which acts as a scaffold; this protein is absent from mitochondria [107]. However, given that BER within mitochondria takes place in the inner membrane where mtDNA is organized into nucleoids, it has been suggested that other nucleoid proteins, such as TFAM, regulate BER pathway within mitochondria as a result of its DNA-binding activity and protein interactions [108]. Of particular interest is the very recent detection of polymerase β (pol β), known as a key nuclear BER protein, in mitochondrial protein extracts from mammalian tissue and cells, contrary to what it has been believed about the “monopoly” of pol γ within mitochondria; indeed, human kidney cells with polβ knock-out exerted higher mtDNA damage [109].

The only repair pathway not yet identified in the mitochondria is the NER pathway which copes with the repair of bulky DNA adducts [82]. It involves, among others, the repair of bulky DNA adducts and cisplatin induced intrastrand crosslinks [9]. NER pathway involves the removal and resynthesis of a short fragment on the damaged strand [97]. To date, the only hint regarding NER pathway in the mitochondria is the localization of the transcription-coupled NER proteins CSA and CSB (Cockayne syndrome proteins) to mitochondria upon oxidative stress [110]. Current belief is that adducts requiring NER for their removal will accumulate into mitochondria resulting to mtDNA mutations and, ultimately, mtDNA degradation [9]. MMR activity and strand break repair (SBR) and double-strand, are also included in the DNA repair repertoire of mitochondria [84,111,112,113]. DNA mismatch repair corrects mis-incorporation and slippage errors introduced by DNA polymerase during replication and base mismatches caused either spontaneously or due to deamination, oxidation, and alkylation [84].

The lack of an efficient MMR and NER systems makes mitochondria vulnerable to the accumulation of miscoding lesions in mtDNA, which in turn can promote mtDNA mutations and block DNA replication [18,114]. Of great importance, pol γ can bypass some lesions by nucleotide incorporation opposite a template lesion and further extension of the DNA primer past the lesion [115]. This process of translesion synthesis is critical for the genetic integrity of mtDNA. Regarding translesion synthesis, PrimPol (primase-polymerase), an archaic enzyme with dual primase and polymerase activities has been identified in human cells; indeed, some PrimPol is present in the nuclear DNA compartment but a larger fraction is located inside mitochondria [116]. Regarding its role within mitochondria, PrimPol exerts synergy with polγ and facilitates replication fork progression by acting as a translesion DNA polymerase or as a specific DNA primase reinitiating downstream of lesions that block synthesis during mitochondrial DNA replication [116,117]. PrimPol has the unique feature ofde novoDNA synthesis solely with dNTPs, and the ability to tolerate lesions such as 8-oxoG and AP (apurinic/apyrimidinic or abasic) sites in DNA.

Similarly, petite integration frequency 1 (Pif1) helicase is a major contributor regarding mtDNA replication. Pif1 localizes to both the nucleus and the mitochondrion; however, its mitochondrial localization in human cells is regulated by alternative splicing, which produces α and β isoforms. Isoform α localizes to the nucleus, whereas the majority of isoform β localizes to the mitochondrion, with some residual nuclear signal [118]. Pif1 is involved in mtDNA maintenance. It contributes to the reduction of DNA strand breaks in mtDNA as well as the repair of UV- and ethidium bromide-damaged mtDNA [119,120]. In addition, Pif1 helicase may assist Twinkleto overcome its inability to unwind G-quadruplex DNA, thus mediating the removal of the genotoxic G-quadruplexes from Twinkle [121]. Recently, it was demonstrated that Pif1 inactivation causes a phenotype of mitochondrial myopathy in mouse, due to deficiency to repair oxidative stress-induced mtDNA damage [122].

Furthermore, there is evidence that mammalian mitochondria may have the ability to directly repair simple alkylations, like methylated or ethylated derivatives [123,124,125]. The mitochondrial genome is known to retain a very low level of DNA methylation [126]. MtDNA can be methylated by machinery existing inside of the mitochondria, thus mediating the control of mitochondrial gene expression [127,128,129,130]. A recent study demonstrated that platelet mtDNA methylation is implicated in the etiology of cardiovascular diseases [126].

Moreover, it has been demonstrated that mammalian mitochondrial protein extracts possess both homologous recombination and non-homologous DNA end-joining activity [131,132]. Indeed, mitochondrial DNA end-binding (DEB) activity is present independently from nuclear DEB activity and is roughly equivalent in potency to that found in the nucleus [133]. The mitochondrial DEB factor shares functional and structural similarities with the nuclear Ku factor, a heterodimer composed of the Ku70 and Ku86 subunits, which is essential for efficient nuclear non-homologous DNA end joining [134]. A mitochondria-specific form of DNA ligase III is responsible for the mitochondrial end-joining activity and is encoded by the same gene that encodes nuclear DNA ligase III [135]. Interestingly, recent evidence suggest that mitochondria lack classical non-homologous DNA end-joining (NHEJ) but possess efficient microhomology-mediated end-joining (MMEJ) activity [136]. Mitochondrial MMEJ depends on ligase III, too. In addition, mitochondria-specific expression of poly (ADP-ribose) polymerase 1 (PARP-1), CtBP interacting protein 1 (CtIP), FEN1 (flap endonuclease 1), meiotic recombination 11 (MRE11), and RAD50 are also involved in mitochondrial MMEJ [136]. A minimum length of 5-nt microhomology was necessary for efficient MMEJ in mitochondria. Respectively, the efficiency of the MMEJ is improved when the length of the microhomology region is increased and when the microhomology region is more proximal to the DNA terminus of the double-strand break [136].

Deletion of mtDNA represents the most serious damage to the mitochondrial genome and may be produced during the repair of double-strand breaks [137,138,139,140]. Unfortunately, recombination errors could have important implications for mtDNA integrity in aged cells. It has been suggested that, in aged cells, mitochondrial dysfunction increases the production of ROS, thus activating a recombination mechanism of mtDNA, which in turn, if hyper-activated, increases the overall recombination errors, mtDNA deletions, ROS production and ultimately, damages mitochondria, through a vicious cycle model [141].

## 4. Heteroplasmy, Mitochondrial Dynamics, and Mitophagy

The third level of defense regarding mtDNA integrity, and therefore organelle homeostasis is the condition of heteroplasmy that characterizes mitochondria; mtDNA exists in numerous identical copies per cell in mammals (homoplasmy), but can also exist in multiple forms within the same tissue or cell (heteroplasmy) [142].Typically, approximately 1000 copies of mtDNA are present per cell, although the actual number may vary depending on the cell type [43]. Given that each cell has many mitochondria, it is concluded that a significant amount of mitochondrial genome is located within each cell, a fact of great importance regarding the accumulation of mutations in mtDNA [143]. Literally speaking, heteroplasmy does not offer protection against mtDNA instability per se, but, most importantly, for the organelle’s genomic stability as a whole. According to Wallace, there is a threshold regarding the number of accumulated mutations in mtDNA that define the transition to a disease state [143]. This threshold depicts the potential of the remaining wild-type mtDNAs within the organelle to compensate for the genetic defect of mutant molecules [3]. After reaching this threshold, mitochondria become dysfunctional, and at this point mechanisms regarding mitochondrial dynamics and clearance are employed.

Mitochondrial dynamics is a term used to describe two main macroscopic behaviors of these organelles: fusion (the joining of two organelles into one) and fission (the division of a single organelle into two), whereas mitochondrial clearance refers to mitophagy, a selective degradation of mitochondria via the autophagic pathway. The maintenance of the ideal balance between these two dynamic mitochondrial behaviors is of paramount importance [144]; derangement of the normal balance in either direction may result in the accumulation of damaged mitochondrial genome and dysfunctional organelles [145]. Multiple post-translational modifications are involved in the regulation of mitochondrial fusion and fission [146,147].

Mitochondrial fusion is primarily orchestrated by large dynamin-related GTPases termed mitofusins (MFN/ MFN1 and MFN2), plus Opa1. Fusion enables mitochondria to distribute mtDNA to all parts of the cell and to replenish damaged mitochondrial DNA [148,149,150]. Dilution of dysfunctional enzymes and mutated mtDNA is considered to be a repetitive process for dysfunctional mitochondria [122]. Fission is mediated by the mitochondrial fission proteins: dynamin-related protein 1 (Drp1); mitochondrial fission 1 protein (FIS1);mitochondrial fission factor(Mff);and mitochondrial dynamics proteins of 49 and 51 kDa (MiD49/51) and is required for cell division and the removal of damaged mitochondria by mitophagy [149,150]. Mitochondrial fission is a major process of cell homeostasis: it distributes mitochondria along cytoskeletal tracks, contributes to the equal distribution of mitochondria into daughter cells during mitosis, and facilitates the removal of dysfunctional mitochondria by partitioning the damaged components to a daughter organelle that can be recovered by fusing with healthy mitochondria or targeted for degradation by mitophagy [146]. Interruption of either fusion, fission, or mitophagy due to loss-of-function mutations in the respective mitochondrial proteins disrupts mitochondrial quality control system, causes instability in mtDNA, and provokes compensatory mitochondrial proliferation, thus inducing neurodegenerative, cardiovascular, and other age-related diseases [149].

**Figure 1:**
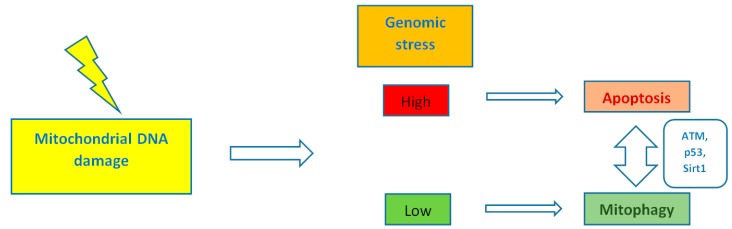
The level of mitochondrial DNA damage determines whether mitophagy or cell death will be stimulated. ATM (ATM serine/threonine kinase), p53 and Sirt1 (sirtuin 1) mediate the signaling between the nucleus and the mitochondria.

**Figure 2:**
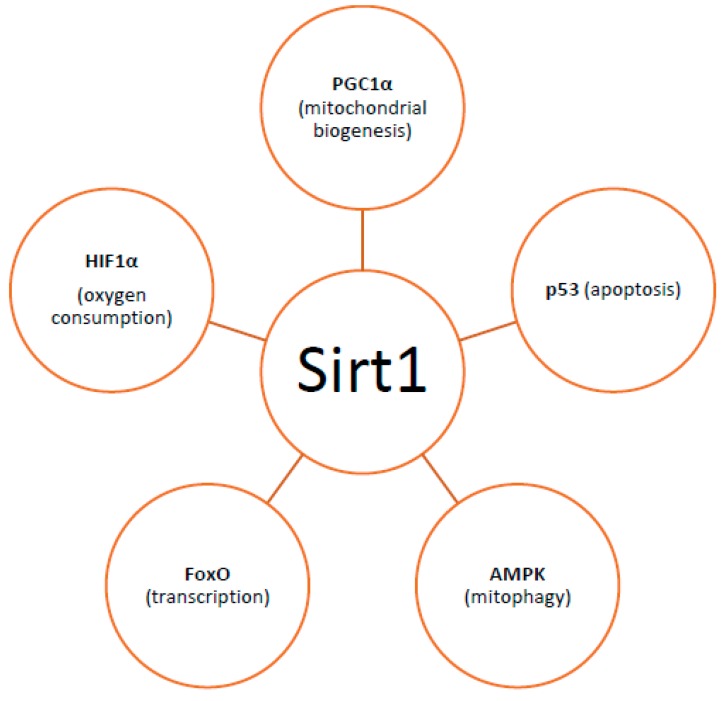
Factors with which Sirt1 interacts regarding mitochondrial homeostasis. [PGC1a: peroxisome proliferator-activated receptor gamma co-activator (PGC1a), HIF1a: hypoxia-inducible factor 1-alpha, AMPK: 5′ adenosine monophosphate-activated protein kinase].

By controlling both apoptosis, which is mediated by Bcl2 (B-cell lymphoma 2) family mitochondrial death proteins, and necrosis, which is mediated by opening of the mitochondrial permeability transition pore, mitochondria regulate cell fate [151]. Importantly, whether DNA damage insults stimulate mitophagy or cell death, is a matter depending on the grade of DNA damage; low levels of DNA damage stress may induce mitophagy instead of apoptosis, whereas high levels of genomic stress stimulate apoptosis and inhibit mitophagy [152]. The crosstalk between mitophagy and apoptosis is mediated through three major nucleus-to-mitochondria signaling pathways linked to genomic stability and mitochondrial homeostasis [152]: (1) the protein kinase ATM (ATM serine/threonine kinase);(2) the tumor suppressor p53; and (3) sirtuin1 (Sirt1) (Figure 1). ATM is a major activator of the DNA damage response which at low levels of double-strand breaks it promotes cell survival, while in case of excessive double-strand break damage, it triggers apoptosis [153]. Similarly, under lethal genotoxic insult, p53 transcriptionally upregulates pro-apoptotic proteins, whereas under low levels of stress it promotes pro-autophagy proteins, thus inducing mitophagy [154]. Of note, ATM functions partly through p53 stabilization. ATM mediates the phosphorylation of MDM2 (murine double minute 2) protein (an E3 ubiquitin ligase); the phosphorylated MDM2 lacks the ability to poly-ubiquitinate p53, thus leading to its stabilization [155]. Except for the direct phosphorylation of MDM2, ATM mediates both the direct phosphorylation of N-terminal serine-15 residue of p53 and indirectly regulates serine-20 phosphorylation by controlling activation of the checkpoint kinase chk2, thus impeding the interaction of p53 with MDM2 and, therefore increasing the stability of the p53 protein and activating its transcriptional activity [156]. Sirt1 belongs to the family of mammalian class III histone deacetylases [157]. It is primarily a nuclear protein; however, Sirt1’s deacetylation of PGC1α has been extensively implicated in metabolic control and mitochondrial biogenesis. Sirt1 interacts with PGC1a, which is the master regulator of mitochondrial biogenesis; caloric restriction and energetic status that elevates Sirt1 levels or NAD^+^ (nicotinamide adenine dinucleotide) levels could promote the accumulation of PGC-1α in the nucleus, thus resulting in the transcription of genes that are necessary for mitochondrial function and biogenesis [157]. Furthermore, Sirt1 inhibits apoptosis after excessive toxic stress through inactivation of p53, and interacts with AMPK (5′ adenosine monophosphate-activated protein kinase), a key regulator of metabolism and energy homeostasis, in order to stimulate mitophagy [158,159]. Additionally, Sirt1 deacetylates members of the Forkhead box O (FoxO) transcription factor family, particularly in response to stress [160]. Moreover, Sirt1 deacetylates and represses the activity of hypoxia-inducible factor 1-α (HIF1α) which is known to down-regulate mitochondrial function and oxygen consumption by inducing pyruvate dehydrogenase kinase 1 (PDK1) [161,162] (Figure 2).

## 5. Conclusions

About 30 years ago, two reports suggested for the first time that deletions and point mutations of mitochondrial genome could be pathogenic [163,164]. Since then, a growing body of evidence has demonstrated the direct or indirect association between mitochondrial dysfunction and the pathogenesis of many neurodegenerative, cardiovascular, and metabolic diseases, as well as cancer and ageing. The hallmark which provokes the pathogenic cataract seems to be the failure of mitochondrial quality control systems, particularly the disruption of mitochondrial genome integrity. Mutations in mtDNA are generated due to spontaneous errors of DNA replication or through unrepaired damage to mtDNA that introduces mis-coding lesions. In addition, it has been demonstrated that except for mtDNA mutations, a reduction in the mtDNA copy number can be pathogenic too [165,166]. Indeed, mitochondrial dysfunction can arise not only from mutations and damage directly related to mitochondrial genes, but also from mutations in nuclear genes that encode proteins which translocate to the mitochondria and are necessary for the biogenesis and function of these multifaceted organelles, as well as from the impairment of nucleus-to-mitochondria signaling which refers to the ability of both the nuclear and mitochondrial genome to communicate and function synergistically [152,167]. More intriguingly, mtDNA repair itself may promote disease through the formation and accumulation of large deletions or recombination of mtDNA [168].From an opposite point of view, mitochondria can also promote genotoxic stress, thus affecting nuclear genome integrity, through the production of metabolic byproducts such as ROS or lipid peroxidation products [83].

Mitochondrial genome instability is associated not only with genetic defects in mtDNA or in nuclear genes encoding proteins that function in the mitochondria, but also with genes involved in supplying the mitochondrial nucleotide precursors needed for DNA replication [21,169]. The dNTPs represent the building blocks for DNA replication, and, as far as mitochondria are concerned, dNTPs pool arise either through active transport of cytosolic dNTPs or through the purine and pyrimidine salvage pathways by action of two mitochondrial deoxyribonucleoside kinases, thymidine kinase 2 (TK2), and deoxyguanosine kinase (DGUOK) [169]. Mutations in the genes that encode these enzymes involved in the salvage pathway cause several forms of mtDNA depletion syndromes [168]. In post-mitotic tissues, mtDNA replication is the major user of cytoplasmic dNTPs, thus forcing the burden of mitochondrial dNTP pool synthesis on the two mitochondrial deoxyribonucleoside kinases [170]. In contrast to the mitochondrial salvage pathway, nucleotide precursors required for DNA replication can be directly obtained by reduction of ribonucleoside diphosphates to deoxyribonucleoside diphosphates by ribonucleotide reductase, which is made up of two subunits, a large one (R1 subunit) and a smaller one (R2 subunit) [169]. Cells have two forms of the R2 subunit, a cell cycle regulated form that is maximally expressed in S-phase, and a p53-inducible form known as p53R2 [169]. The p53R2 form is required for a basal level of DNA repair and de novonucleotide synthesis in non-proliferating cells; it catalyzes the biosynthesis of deoxyribonucleotides by storing organic free radicals required for catalysis in the R2 subunit [170]. Mutations in the *RRM2B* (ribonucleoside-diphosphate reductase subunit M2 B) gene, encoding the p53-controlled ribonucleotide reductase subunit, has been implicated in the pathogenesis of mtDNA depletion syndrome [171].

**Figure 3:**
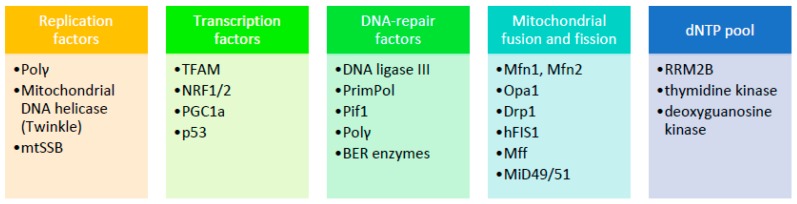
Summary of factors mentioned in the text that are associated with instability of mammalian mitochondrial DNA. The list presented herein is not exhaustive. (Polγ: polymerase γ, mtSSB: mitochondrial single stranded DNA binding protein, TFAM: mitochondrial transcription factor A, NRF1/2: nuclear respiratory factors 1 and 2, PrimPol: primase-polymerase; Pif1: petite integration frequency 1, BER: base excision repair, Mfn: mitofusin, Opa1: optic atrophy protein 1,Drp1: dynamin related protein 1,hFIS1: fission 1 protein, Mff: mitochondrial fission factor, MiD49/51: mitochondrial dynamics proteins of 49 and 51 kDa, RRM2B: ribonucleoside-diphosphate reductase subunit M2 B).

**Figure 4:**
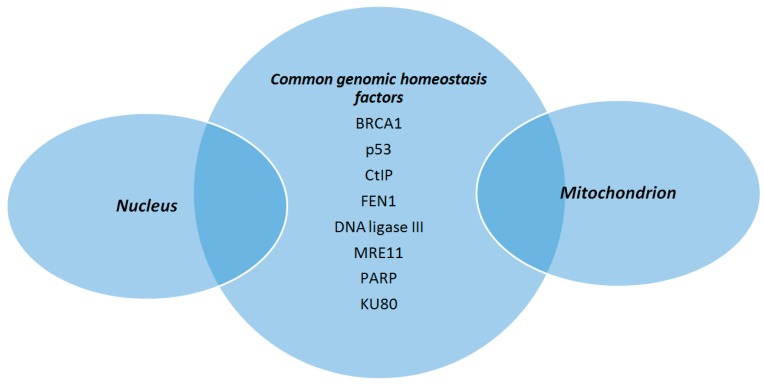
Proteins involved in the maintenance of mitochondrial DNA integrity, in addition to their role in ensuring nuclear genomic homeostasis. (BRCA1: breast cancer type 1 protein, CtIP: CtBP-interacting protein, FEN1: flap endonuclease 1, MRE11: meiotic recombination 11, PARP: poly (ADP-ribose) polymerase).

The scope of this review is to highlight basic aspects of mitochondrial genome structure and DNA repair mechanisms present within mitochondria and how these features contribute to the maintenance of mtDNA integrity (Figure 3). Of great importance, a number of nuclear genome maintenance factors have been described as having also a mitochondrial location and are involved in mtDNA protection (Figure 4). In case of failure to restore or bypass troublemaking DNA damage, mitochondria exert an extra line of defense in order to retain cell homeostasis, which is basically relevant to mitochondrial dynamic behaviors. The identification of physiological regulators of mtDNA protection and repair mechanisms may open the way for the development of novel therapeutic strategies aiming to restore mitochondrial dysfunction which is an important component of various disease states. In this regard, thyroid hormone (TH) is shown to enhance mitochondrial function and reduce cellular oxidative stress in patients with mtDNA defects [172]. TH has been shown to stimulate mitochondrial biogenesis and increase mitochondrial mass, mitochondrial protein synthesis, and mtDNA content in both physiological and pathological conditions via induction of TFAM, PGC1a, and specific mitochondrial thyroid hormone receptors [173]. As mentioned above, especially *TFAM*, may also serve as a shell for mtDNA to enhance its resistance to genotoxic insults. From another point of view, it is interesting to note that TH may inhibit the genotoxic stress that is produced by mitochondria during myocardial ischemia and leads to apoptosis and heart failure [174,175]. Furthermore, TH was recently shown to induce selective mitophagy of damaged mitochondria blocking DNA damage and protecting hepatocytes from carcinogenesis [176].
